# Using Acoustic Speech Patterns From Smartphones to Investigate Mood Disorders: Scoping Review

**DOI:** 10.2196/24352

**Published:** 2021-09-17

**Authors:** Olivia Flanagan, Amy Chan, Partha Roop, Frederick Sundram

**Affiliations:** 1 Department of Psychological Medicine Faculty of Medical and Health Sciences University of Auckland Auckland New Zealand; 2 School of Pharmacy Faculty of Medical and Health Sciences University of Auckland Auckland New Zealand; 3 Faculty of Engineering University of Auckland Auckland New Zealand

**Keywords:** smartphone, data science, speech patterns, mood disorders, diagnosis, monitoring

## Abstract

**Background:**

Mood disorders are commonly underrecognized and undertreated, as diagnosis is reliant on self-reporting and clinical assessments that are often not timely. Speech characteristics of those with mood disorders differs from healthy individuals. With the wide use of smartphones, and the emergence of machine learning approaches, smartphones can be used to monitor speech patterns to help the diagnosis and monitoring of mood disorders.

**Objective:**

The aim of this review is to synthesize research on using speech patterns from smartphones to diagnose and monitor mood disorders.

**Methods:**

Literature searches of major databases, Medline, PsycInfo, EMBASE, and CINAHL, initially identified 832 relevant articles using the search terms “mood disorders”, “smartphone”, “voice analysis”, and their variants. Only 13 studies met inclusion criteria: use of a smartphone for capturing voice data, focus on diagnosing or monitoring a mood disorder(s), clinical populations recruited prospectively, and in the English language only. Articles were assessed by 2 reviewers, and data extracted included data type, classifiers used, methods of capture, and study results. Studies were analyzed using a narrative synthesis approach.

**Results:**

Studies showed that voice data alone had reasonable accuracy in predicting mood states and mood fluctuations based on objectively monitored speech patterns. While a fusion of different sensor modalities revealed the highest accuracy (97.4%), nearly 80% of included studies were pilot trials or feasibility studies without control groups and had small sample sizes ranging from 1 to 73 participants. Studies were also carried out over short or varying timeframes and had significant heterogeneity of methods in terms of the types of audio data captured, environmental contexts, classifiers, and measures to control for privacy and ambient noise.

**Conclusions:**

Approaches that allow smartphone-based monitoring of speech patterns in mood disorders are rapidly growing. The current body of evidence supports the value of speech patterns to monitor, classify, and predict mood states in real time. However, many challenges remain around the robustness, cost-effectiveness, and acceptability of such an approach and further work is required to build on current research and reduce heterogeneity of methodologies as well as clinical evaluation of the benefits and risks of such approaches.

## Introduction

### Background

Mood disorders are common with 12-month prevalence rates ranging between 6.6% and 11.9% [1] and represent significant personal, social, and economic burden [2,3]. However, these disorders remain underrecognized and undertreated [4]. Early objective identification of warning signs that are associated with such disorders can facilitate time-sensitive interventions and early responses by the health care provider [5]. However, current methods of mental health assessment are limited in their capacity to accomplish this due to the following reasons. First, mental health assessments rely on self-reporting and clinical interviews, which depend on individuals’ memories and consequently are susceptible to recall and other biases [6]. Second, assessments often take place in clinical contexts by health care professionals, which may be substantially different from one’s usual environment and thus limits ecological validity [7]. Finally, individuals may not recognize the need to seek treatment until symptoms reach a level of severity that warrants clinical attention, making treatment more difficult than if the mood changes had been detected earlier [8]. Moreover, the COVID-19 pandemic is having a profound impact on our way of life and mental well-being [9-11]. Increased fear, uncertainty, and anxiety as well as the public health measures taken to manage the pandemic and social/economic crisis mean that people are more vulnerable to developing mood disorders and engagement with health care providers is even more difficult [12]. There is thus a need for better tools, which can provide objective mental health assessments on an ongoing basis and within a home setting, to enable earlier and accurate diagnosis of mood disorders and detection of changes in mental state.

There has been increasing interest in the use of data-driven approaches in the detection and monitoring of health and disease [13,14]. The rapid growth of smart-sensor integration in smartphones allows the collection of objective quantitative markers of behavior and function [15,16]. In mental health, this approach may be particularly feasible; for example, speech can be a key distinguishing characteristic for the diagnosis and monitoring of mental health disorders [17]. In this regard, diagnosis and monitoring are overlapping concepts as changes to mental state can be monitored and therefore prospectively tracked for diagnostic purposes. Current clinical measures such as the Young Mania Rating Scale for the diagnosis of mania [18] and the Hamilton Depression Scale for depression [19] both use clinical observations of speech to aid diagnosis. In bipolar disorder (BD), pressure of speech is a key diagnostic symptom in mania, and poverty of speech in depression. Evidence suggests that these speech differences can be quantified via measurement of verbal fluency (eg, word and error count, switching, and clustering abnormalities) [20]. With the emergence of machine learning approaches [21], the automatic classification of speech as an objective measure for mood disorders is becoming more feasible. Smartphones may therefore offer a unique opportunity to augment current mental health assessment methods or bypass many of the limitations associated with them [22].

In this review, speech/voice patterns or features refer to measurable and objective aspects of speech that affect the acoustic quality of speech production (eg, prosodic features such as pitch). The reader is referred to the review by Malhi et al [23] which covers several aspects of these features. Classifiers can be used to investigate mood states, whereby a classifier refers to a hypothesis or discrete-valued function that is used to assign (categorical) class labels to particular data points [24]. Studies have classified people according to presence/absence, severity, or score-level prediction based on brain, wearable, and Twitter activity using machine learning [25-27]. However, the well-established relationship between voice and mood disorders [25] has been under-investigated—the emergence of machine learning approaches [21] leads to the question of whether smartphone voice data could provide clinical insight into mood symptoms in real time. In recent years, studies have discussed the promise of smartphone voice data to diagnose mood disorders [28,29]. However, fundamental scientific questions remain before smartphones can be used as validated and objective clinical tools [30]. Although there is an ever-growing number of studies focusing on the collection of objective data from smartphone or external sensors to diagnose and monitor mood disorders, only a small portion of these have included speech features as a key objective marker. Considering the importance of this emerging field, the speed of innovations, and new developments [28], it was our aim to synthesize the literature on the use of speech patterns from smartphones in the diagnosis and monitoring of mood disorders, and the accuracy and technical feasibility of this approach.

### Objectives

The aim of this review was to evaluate the current state of research on the use of speech patterns from smartphones to diagnose and monitor mood disorders. Specifically, objectives of this review are to (1) characterize studies that have been conducted on speech patterns to diagnose and monitor mood disorders using smartphone devices and (2) provide details on the technical feasibilities of smartphones to achieve this, such as their ability to control ambient noise and how privacy was managed. “Speech features and patterns” referred to in this review describe objective markers such as the acoustics of, rather than behavioral patterns collected from smartphone use (eg, the length of time spent on the phone).

## Methods

### Design

**Figure 1:**
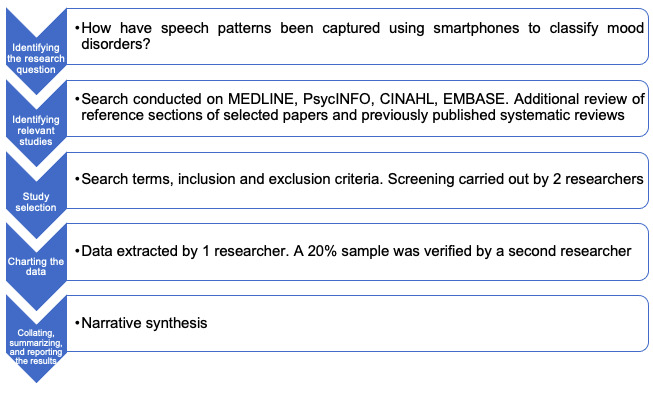
Methodological framework used in this scoping review as per Arksey and O’Malley [32].

A scoping approach was adopted for this review which according to Nicholas and colleagues [31] aims “to map rapidly the key concepts underpinning a research area and the main sources and types of evidence available, and can be undertaken as stand-alone projects in their own right, especially where an area is complex or has not been reviewed comprehensively before.” This method was chosen because the field of machine learning in mood disorders is advancing exponentially; therefore, it was deemed appropriate to focus specifically on exploring broadly the nature of research activity, as per Arksey and O’Malley’s [32] first goal of scoping reviews. This study was guided by the methodological framework proposed by Arksey and O’Malley’s [32] which involves a 5-stage process (Figure 1) that was benchmarked against the PRISMA (Preferred Reporting Items for Systematic Reviews and Meta-Analyses) guidelines [33] to ensure rigor.

### Search Strategy

MEDLINE (ProQuest), PsycINFO (ProQuest), EMBASE (Elsevier), and CINAHL (EBSCO) databases were used to search for studies published from the date of database conception to November 24, 2020. The following search terms and their variants were used in each database: “mood disorders”, “smartphone”, and “voice analysis”, using the Boolean search operator “OR” and “*” where appropriate, and combined using the Boolean operator “AND.” Multimedia Appendix 1 presents the full search strategy. To capture appropriate studies, the search was limited to English language publications only. Textbox 1 describes the full inclusion and exclusion criteria.

**Inclusion and exclusion criteria. IVR: interactive voice response; EMA: ecological momentary assessment.**

**Inclusion criteria**

- Use of smartphone
- Focus on diagnosing/monitoring of a mood disorder including depression, mania, and bipolar affective disorder
- Clinical populations recruited prospectively
- Captures voice data
- English language

**Exclusion criteria**

- Not using a smartphone device (eg, laptop)
- Focus on other health conditions rather than mood disorders (eg, Parkinson disease), or focus on mood disorder treatment/intervention rather than diagnosis/monitoring; or examined effects of speech patterns, or smartphone use in general without reference to mood; or collected IVR/EMA data only
- Does not capture voice data
- Non-English language publications

### Search Outcomes

**Figure 2:**
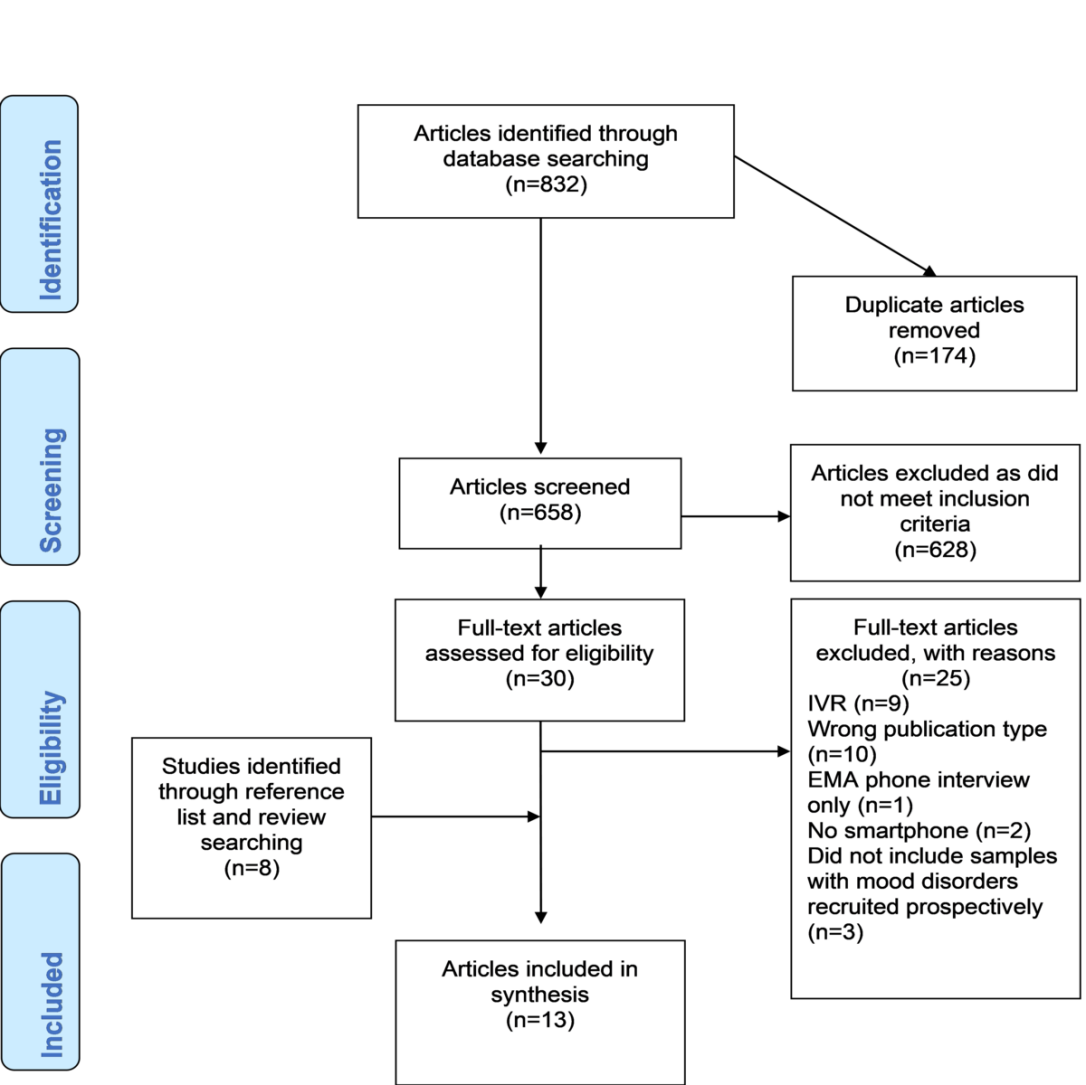
PRISMA flowchart demonstrating search process. EMA: ecological momentary assessment; IVR: interactive voice response; PRISMA: Preferred Reporting Items for Systematic Reviews and Meta-Analyses.

Figure 2 details the process of study selection using the PRISMA flow diagram [33]. After duplicates were removed, articles were downloaded into Rayyan [34], a systematic review web application, where inclusion/exclusion decisions were made. Screening of all titles and abstracts was undertaken by the lead author (OF). A second reviewer, blind to the inclusion/exclusion decisions of the articles, randomly screened 20% of titles and abstracts, with agreement on 128 out of 132 articles (96.9%) screened for inclusion/exclusion and all conflicts resolved by consensus following discussion between both raters. Both OF and the second reviewer read all articles selected for full-text review. Reference lists of articles included in the review were also manually screened to identify any relevant studies that were not identified through database searching, and systematic reviews that were identified during the search process were also screened and relevant studies extracted.

### Data Extraction and Analysis

Data were charted by OF and entered onto a data charting form using Microsoft Excel. To ensure accuracy and consistency of the process, a sample of 20% of the information being entered into Excel was verified by a second reviewer [35]. No significant discrepancies or errors were detected. The charting process allowed the researcher to describe the characteristics of the studies included in the review and prepared studies for analysis [36]. Textbox 2 describes a summary of the data captured from the studies included in the review. To analyze the data, a narrative review synthesis method [37] was selected to capture each study’s characteristics and methods to investigate voice analysis in mood disorder diagnosis and monitoring.

**Summary of data captured.**

**Summary of data captured**

- Author, year, location, study design
- Sample size and diagnosis
- Aim of studies
- Methods of data capture

**Length of audio data capture**

- Timing of data capture
- Type of audio data captured
- Ambient noise control
- Privacy
- Clinical outcome measurement
- Classifier used
- Key findings

## Results

### Characteristics of Included Studies

A total of 13 out of the 832 studies initially identified were included in this scoping review (studies were mostly excluded due to reasons of not involving a smartphone device and lack of focus on monitoring or detecting a mood disorder). The publication year of included studies ranged between 2011 and 2020 with 77% (10/13) of articles published from 2015 onward, highlighting the increase in interest and recent advancements in this area. Included studies used single-arm observational designs [38-45], quasi-experimental designs [46-48], and observational case reports [49,50].

Each study’s aims, key characteristics, and findings are detailed in Table 1. Studies reported a variety of speech features analyzed and these features are summarized in Textbox 3. Additionally, an overview considering overlaps and differences across the included studies is provided covering these aspects: smartphone device/platform/apps and data storage; characteristics of data capture; noise and privacy; and clinical outcome measurement.

**Table 1:** Results of included studies.

Study; year; location; design; sample size and diagnosis	Study aim; methods of data capture; duration; clinical evaluations	Audio data captured; classifier used	Assessment; key finding
Abdullah et al [38]; 2016; USA; single-arm observational design; 7 patients with BD^a^	Assessed stability and rhythmicity for individuals with BD; microphone activated during daily conversations; 4 weeks; ratings via Social Rhythm Metric-5 (SRM-5) via initial questionnaire, poststudy questionnaire, and interview	Speaking rate and variations in pitch; SVM^b^	Classification based on the same individual’s data; classified individuals into stable or unstable mood states with high accuracy (precision=0.85 and recall=0.86).
Dickerson et al [50]; 2011; USA; observational case report; 1 participant with major depressive disorder	Created a real-time depression monitoring system for the home; free speech response to daily questions; 2 weeks; scores from Centre for Epidemiological Studies Depression (CES-D) subjectively provided via touchpad	Fundamental frequency and speech pause time; pitch detection algorithm	Classification based on the same individual’s data; the model fit the data with a residual error of 0.0916 on 12 degrees of freedom (P<.011).
Faurholt-Jepsen et al [39]; 2016; Denmark; single-arm observational design; 28 patients with BD	Investigated voice features collected during phone calls as objective markers of affective states in BD; natural phone calls; 12 weeks; fortnightly clinical interviews using the HAMD^c^ and YMRS^d^	openSMILE toolkit with 6552 numerical features including pitch, variance, etc.; random forest algorithms	Classification based on the same individual’s data; phone calls could classify manic states with an AUC^e^ of 0.89 compared with an AUC of 0.78 for depressive states.
Gideon et al [46]; 2016; USA; quasi-experimental design; 37 patients with BD with rapid cycling	Investigated acoustic variations with different types of phones and the preprocessing and modeling changes necessary to detect mood; natural phone calls; 6-12 months; weekly calls with clinicians to conduct HAMD and YMRS interviews	Spectral power ratio and spectral centroid; SVM	Comparison between the Galaxy S3 and Galaxy S5 groups based on their individualized data; preprocessing, feature extraction, and data modeling improve the performance of mixed device systems (AUCs of 0.57 and 0.64 for manic and depressed states, respectively, to 0.72 and 0.75).
Grünerbl et al [40]; 2015; Austria; single-arm observational design; 10 patients with BD	Introduced a system which can recognize depressive and manic states and detect state changes of patients with BD; natural phone calls; 12 weeks; HAMD and YMRS examinations performed every 3 weeks by clinicians over the phone	Kurtosis energy, mean second and mean third MFCC^f^, mean fourth delta MFCC, maximum ZCR^g^ and mean harmonic-to-noise ratio, SD, and range F0; naïve Bayes, k-nearest neighbors, j48 search tree, and conjunctive rule learner algorithms	Classification based on the same individual’s data; phone call behavior did not provide as high a recognition rate as voice features. A fusion of 4 different sensor modalities achieved the highest recognition accuracies of 76% and state change detection precision and recall of over 97%.
Guidi et al [49]; 2015; France; observational case report; 1 patient with BD	Collected and analyzed prosodic features via an Android app; structured tasks (eg, reading, counting), commenting on a picture performed 15 times; 14 weeks; Quick Depression Inventory (QID) and YMRS assessments conducted by a clinician during the day before each voice recording session	Mean F0, jitter, and F0 SD; the SWIPE algorithm (pitch estimator algorithm)	Classification based on the same individual’s data; mean F0 from each voiced segment can be reliably estimated, but weak correlations were reported between audio features and mood.
Karam et al [41]; 2014; USA; single-arm observational design; 6 patients with BD	Investigated whether speech collected in an unstructured setting can be used to assess underlying mood state; phone calls as well as speech in clinical interviews; 6 months to 1 year; weekly phone-based HAMD and YMRS assessments with a clinician	23 Low-level features extracted using the openSMILE toolkit including pitch; root mean square energy, zero-crossing rate, and the amplitude of the speech waveform; SVM	Classification based on the same individual’s data; hypomania and depression can be differentiated from euthymia using speech-based classifiers trained on both structured and unstructured cell phone recordings.
Maxhuni et al [42]; 2016; Austria; single-arm observational design; 10 patients with BD	Evaluated the performance of several classifiers, different sets of features, and the role of questionnaires for classifying BD episodes; natural phone calls; 12 weeks; scheduled interviews with a clinician by phone every 3 weeks using the HAMD, ADS (Allgemeine Depressionsskala/Common Depression Scale), YRMS, and Mania Self-Rating Scale (MSS)	Features extracted using openEAR and Praat. LOG energy, ZCR, probability of voicing, F0^h^, MFCC, MEL spectrum, spectral energy in bands, spectral roll-off point, spectral flux, spectral centroid, and spectral max and min with discrete Fourier transform; comparison of C4.5, random forest, SVM, k-nearest neighbors, naïve Bayes, AdaBoost, and bagging algorithms	Classification based on the same individual’s data; classification accuracy using spectral characteristics=82% or emotional characteristics=82%. Decision trees performed best.
Muaremi et al [43]; 2014; Austria; single-arm observational design; 12 patients with BD	Explored the feasibility of voice analysis during phone conversation to predict BD episodes; natural phone calls; 12 weeks; HAMD and YMRS interviews conducted by a clinician every 3 weeks at the hospital	openSMILE; Kurtosis energy, mean second MFCC, mean third MFCC, mean fourth delta MFCC, maximum ZCR, mean harmonic-to-noise ratio, F0 SD, range F0; comparison of SVM, logistic regression, random forest, and neural networks	Classification based on the same individual’s data; classification accuracy using patient voice characteristics=80%. Combination of all data streams=83%. Random forest algorithms performed the best.
Osmani [44]; 2015; Italy; single-arm observational design; 9 patients with BD	Investigated whether data from smartphone sensors could be used to recognize BD episodes and to detect behavior changes that could signal the onset of an episode using objective, sensor data; natural phone calls; 12 weeks; every 3 weeks by a clinician using the HAMD and YMRS	Phone calls and sound analysis; comparison of naïve Bayes, k-nearest neighbors, search tree, and a conjunctive rule learner algorithm	Classification based on the same individual’s data; sound analysis accuracy=70%, recall=60%, precision=59%. Best accuracy was achieved through a combination of modalities (accelerometer, location, phone and sound recall=97.4%, precision=97.2%).
Pan et al [47]; 2018; China; quasi-experimental design; 21 hospitalized patients with BD	Compared the accuracy of SVM and GMM in the detection of manic state of BD of single patients (smaller sample size) and multiple patients (larger sample size); free open phone calls with clinicians; 2 days; Bech–Rafaelsen Mania Rating Scale (BRMS) used by a clinician while patients were in the hospital	openSMILE; pitch, formants, MFCC, LPCC^i^, gammatone frequency cepstral coefficients, etc. were preprocessed and extracted; comparison of SVM and GMM^j^	Comparison between single-patient experiments (n=3) and multiple patients experiments (n=21) based on their individualized data; LPCC demonstrated the best discrimination efficiency. The accuracy of manic state detection for single patients was better using SVM than GMM methods. Detection accuracy for multiple patients was higher using GMM than SVM methods.
Place et al [45]; 2017; USA; single-arm observational design; 73 patients with a symptom of post-traumatic stress disorder or depression	Reported on models of clinical symptoms for post-traumatic stress disorder and depression derived from a scalable mobile sensing platform; daily audio diary entries; 12 weeks; baseline questionnaire at initial visit, at the end of the study patients completed a semistructured clinical interview on-site with a trained clinician, and completed a close-out survey	Mean speaking fraction, mean speaking rate, mean harmonicity, SD of harmonicity, mean vocal effort, SD of vocal effort, mean pitch variation, SD of pitch variation; comparison of 5 different algorithms for speaking fraction, speaking rate, harmonicity, vocal effort, and pitch variation	Classification based on the same individual’s data; depressed mood was predicted from audio data with an AUC of 0.74.
Gideon et al [48]; 2020; USA; quasi-experimental design; 51 patients with BD	Expanded clinical mood monitoring to predict when interventions are necessary using an anomaly detection framework; natural phone calls and phone calls with clinicians; 6-12 months; calls with clinicians using the HAMD and YMRS to retrospectively rate their mood each week	Emotion features (eg, Mel Filter Banks) using MADDoG^k^ and transcript features (eg, speaker timing); automatic speech recognition model	High YMRS or HDRS compared with personal baseline; TempNorm can be used to transform the symptom severity ratings to effectively predict if an intervention should occur. Transcript features performed best for the clinical calls, while both transcript and emotion features worked well for natural speech.

^a^BD: bipolar disorder

^b^SVM: support vector machine

^c^HAMD: Hamilton Depression Rating Scale

^d^YMRS: Young Mania Rating Scale

^e^AUC: area under the curve

^f^MFCC: Mel-frequency cepstral coefficient

^g^ZCR: zero crossing rate

^h^F0: fundamental frequency

^i^LPCC: linear prediction cepstral coefficients

^j^GMM: Gaussian mixture model

^k^MADDoG: multiclass adversarial discriminative domain generalization

**Most common features used within included studies to analyze vocal aspects of speech.**

**Prosodic features**

These include pitch (F0), speaking rate, jitter, shimmer, loudness, harmonic-to-noise ratio (HNR), log of energy, and Teager energy operation (TEO). Prosodic features represent the long-time (phoneme level) variations in perceived intonation, stress, and rhythm of speech.

- F0 refers to rate of vocal fold vibration.
- Jitter refers to the short-term fluctuations in pitch.
- Shimmer refers to the period-to-period variability of the signal peak-to-peak amplitude.
- Loudness refers to the intensity of auditory sensation produced.
- HNR refers to the average ratio of harmonic energy to inharmonic energy in a voice signal.
- Log of energy refers to the logarithmic short-term energy within a frame.
- TEO refers to amplitude and frequency modulations of vocal tract resonances generated by nonlinear airflows within the vocal tract.

**Spectral and cepstral features**

These include spectral flux (SF), spectral centroid (SC), Mel-frequency cepstral coefficients (MFCCs), linear prediction cepstral coefficient (LPCC), and gammatone frequency cepstral coefficients (GFCCs). These features characterize the speech spectrum, the average sound spectrum for the human voice.

- SF refers to the measure of the amount of frame-to-frame variance in the spectral shape.
- SC is a measure to characterize a spectrum.
- MFCCs are based on the Mel Filter Bank and describe the overall shape of a spectral envelope.
- LPCC models the human vocal tract as an infinite impulse response system that produces the speech signal.
- GFCCs are based upon the Gammatone Filter Bank where the filters model physiological changes in the inner ear and middle ear.

### Smartphone Device/Platform/Applications and Data Storage

The majority of studies provided participants with an Android smartphone as a data collection tool. Dickerson et al [50] provided their participants with an iPhone and Faurholt-Jepsen et al [39] allowed study participants to use their own Android smartphone or were offered to loan an Android smartphone. To facilitate the collection of audio data, all studies, except Pan et al [47], used a cloud database. A variety of downloaded mobile apps were used, such as MoodRhythm [38], Empath [50] MONARCA [39,40,42-44], PRIORI [41,46,48], PSYCHE [49], and a Defense Advanced Research Projects Agency–funded app [45]. In most studies, data were captured locally on the device and then securely transmitted to a server periodically [38,39,41-43,45,46,48-50]. One study temporarily stored data locally on the phone and then uploaded data to the cloud when the phone was being recharged and connected to Wi-Fi [47]. Grünerbl et al [40] stored data on an SD (secure digital) card at the end of everyday (for data security issues), while Osmani [44] did not mention how data were stored in his study.

### Characteristics of Data Capture

#### Length of Audio Data Capture

The length of time spent capturing audio data ranged from 2 days [47] to 12 months [48].

#### Methods of Data Capture

Audio data were captured from participants when they read, counted, or commented on a picture aloud [49]; during daily conversation [38]; natural phone calls [39,40,42-44,46,48,49]; phone calls with clinicians [41,47,48]; daily audio diary entries [45]; or from responding to questions aloud such as “How was your day today” [50]. The frequency of evaluations varied greatly between studies; for instance, studies collected data daily [38-41,43,44,47,50], weekly [45,46,48,49], or were dependent on when phone calls were made [42].

#### Timing of Data Capture

Data were captured either during an acute episode of BD [38-41,43,44,48,49] or depression [50]; or in one study during the daily life of veterans with symptom(s) of post-traumatic stress disorder (PTSD) or depression [45].

#### Audio Data Captured

See Textbox 3 for a description of common audio features captured. Some studies also made use of feature extractors for signal processing and machine learning applications such as openSMILE [40,41,43,47], openEAR, and Praat [42].

### Noise

Only one-third of the included studies referred to a method to control for ambient noise. The methods varied and included using energy intensity and distribution likelihood [38], using a “guard zone”/threshold to filter out noise [50], using a segmentation algorithm robust to variation in noise [46], and using a double-layer sound-insulated glass room when talking [39]. Gideon et al [48] stated that their data consisted of unconstrained natural speech in the presence of noise, so imperfect transcriptions were expected (evident by the 39.7% word count error). However, they note that their previous work reveals that mood recognition (especially mania) is improved by addressing variability in clinical recordings due to device differences [46].

### Privacy

In terms of protecting participants’ privacy, no study evaluated speech content—only speech features were evaluated. Four studies did not report on the measures taken to protect participant privacy [42,44,48,50].

### Clinical Outcome Measurement

Most studies used the Hamilton Depression Rating Scale [39-44,46,48] and the Young Mania Rating Scale [39-44,46,48,49] for assessment of mood. Studies also used the Social Rhythm Metric [38], the Centre for Epidemiological Studies Depression Scale [50], the Quick Inventory of Depressive Symptomatology [49], the Bech–Rafaelsen Mania Scale [47], the Structured Clinical Interview for DSM-5 [45], Primary Care PTSD Screen for DSM-5 [45], and the Patient Health Questionnaire-2 [45].

## Discussion

### Principal Findings

This scoping review evaluates the current state of research on the use of speech patterns from smartphones to diagnose and monitor mood disorders. We found robust evidence that demonstrates a high potential to use smartphone voice data to monitor/detect mood disorders in individuals in real time. These voice analyses can be used to detect changes in mood at the different stages of mental health presentation [25]—first at the onset where acute changes in speech patterns can occur and during remission, as speech patterns return to the individual’s baseline level; and then later to monitor for early warning signs that may predict relapse [39]. There is also potential for these voice data to be used to distinguish between clinical conditions such as BD and schizophrenia [51], and within a disorder, between different clinical states such as mania, hypomania, and mixed states for bipolar [20,46]. This section discusses the key findings from this review (most common speech features, classifiers, and audio capture methods used and smartphone device technical considerations) and the various challenges that remain with regard to accuracy, feasibility, and practical considerations and identification of gaps and future research implications.

### Accuracy, Feasibility, and Practical Considerations

With regard to feature extraction, there are many speech features that have been found to be related to depression and BD [52]. Within the included studies, the most common speech features analyzed included prosodic (fundamental frequency, speaking rate, and energy), spectral (spectral centroid), and cepstral features (Mel-frequency cepstral coefficients). Karam et al [41] revealed that the most informative features for classification of bipolar states are the average binary voiced activity detection, SD of pitch, segment average of the zero-crossing rate, and segment average of the smoothed voiced activity detection. Muaremi et al [43] showed that the most important speech features for prediction of bipolar states were harmonic-to-noise ratio (HNR) value, the number of short turns, and the variance of pitch F0. Moreover, Pan et al [47] found that linear prediction cepstral coefficient and gammatone frequency cepstral coefficient contain important mood information for manic state than other features. Overall, all studies analyzed prosodic features of speech, with F0 being the most common feature. However, due to the natural variations in individual speaking styles and the wide clinical profile of BD and depression, a single-dimensional prosodic feature does not contain sufficient discriminatory information for use as a clinical marker, and a multivariate approach is required. In addition, further research is required to verify whether other features, such as glottal features, can be utilized to monitor and diagnose mood disorders.

Given the current lack of a reliable speech feature or clarity around multivariate features for mood disorder classification or prediction, fusion of objective data measures acquired from multiple sensors (eg, GPS, voice, and acceleration) or a combination of physiological (eg, heart rate variability) and behavioral parameters is a promising approach moving forward. This is reflected in the current work whereby studies that combined data on voice features with other automatically generated objective data increased the accuracy, sensitivity, and specificity of classifying affective states [39,40,43,44].

The 2 most popular modeling and classification techniques include support vector machines (SVMs) and Gaussian mixture models (GMMs). The most common classifier used in this study was SVM [38,41,46,47]. For instance, Pan et al [47] compared SVM with GMM in the detection of a manic state associated with BD of individual and multiple patients. They found SVM provided an appropriate tool for detecting manic states for individual patients, whereas GMM worked better when detecting manic states for multiple patients. Studies that have also compared multiple classifiers [40,42-45] found high promise for the use of random forest and other decision tree classification models in the detection of mood disorders [42,43]. The majority of studies reviewed in this study utilized supervised classification techniques [38,41,46,47] (ie, learning from labeled data to predict the class label of unlabeled input data [53]) rather than other machine learning techniques. This is most likely a result of the focus being on detection and diagnosis. Although SVM and GMM have been widely utilized, results hold promise for decision tree classification methods, which are able to assess the importance of the variables during the training process. This knowledge helps us to discover which nonrelevant parameters can be ignored, potentially resulting in a reduced computational effort on the smartphone.

Included studies in this review mostly used Android smartphones, which is unsurprising given their global market dominance [54]. However, despite their popularity, previous research has indicated less acoustic signal conformity in Android devices [55], attributed to the nonstandard hardware and software designs across manufacturers. Included in this review, Gideon et al [46] compared 2 different phones with various amounts of clipping, loudness, and noise and described methodologies to use during preprocessing, feature extraction, and data modeling to correct these differences and make the devices more comparable. Such methods were found to significantly increase the performance of mixed device systems. Given the increasing global popularity of smartphones, proper processing of acoustic data from multiple types of smartphones will be necessary to increase reliability and accuracy and mitigate the effects of differing amounts of clipping, loudness, and noise. This finding has important implications for engineers who create speech-based mood classification systems for smartphones, as they will have to optimize their design for a wide number of handset models.

In terms of what audio data were captured, the methods varied between using fixed or spontaneous speech. However, the evidence suggests that spontaneous speech such as free conversation or interviews contain more variability and can increase depressive and manic mood-state detection accuracies than using fixed speech (eg, reading text) [52,56,57]. Speech collection in natural environments highlights the applicability for autonomous ecologically valid monitoring of mood disorders. Future research therefore would benefit from adopting an unscripted setup, which preserves naturally expressed emotion. The length of data collection within the studies included in this review varied but were mostly of short duration, resulting in some studies having to exclude participants from final analyses as they did not exhibit recognizable changes in mood state [38,43]. To identify individual patterns that predict state changes, longer monitoring durations (greater than 12 weeks) may be required.

### Gaps and Future Work

Spontaneous speech brings a greater need to handle ambient noise. Less than half of the studies included in the review described how noise was handled. The most practical method used was a “guard zone”/threshold to filter out noise [50] or the use of an algorithm that is robust to noise variation [46]. Future research needs to compare and investigate robust features and modeling techniques to mitigate the effects of noise. For example, a recent study by Mitra et al [58] found that using suitable and robust features and modeling strategies mitigated the performance degradation from varying background conditions. In their case they used damped oscillator cepstral coefficients instead of standard Mel-frequency cepstral coefficients and compared support vector regression and artificial neural networks for depression score prediction, revealing artificial neural networks to be more robust to support vector regressions.

Future research will also need to address technical, acceptability, and ethical issues of smartphone-based monitoring in order for this method to be reliably used in clinical practice. For instance, technical factors such as battery lifetime or individual usage (some individuals bring their smartphones everywhere they go, others do not) of the smartphone may serve as obstacles. Similarly, ethical issues remain such as how an individual’s privacy is preserved, how to mitigate the acceptability concerns (eg, unease or increased anxiety that constant surveillance and monitoring may cause), and how sensitive data concerning mental health are protected. None of the studies included in this review collected data on speech content but only speech features; however, if these systems are to be used in routine clinical care, a high standard of protection from security breaches is required.

Lastly, it is important that future research investigates which combination of speech features are the most accurate for diagnostic and monitoring purposes. Cummins et al [59] have called for greater research collaboration and cooperation in order to progress the field, and more recently, Barnett et al [60] have called for a complete and comprehensive data platform to capture the breadth of available sensor data in a meaningful way. Moving in these directions to find valid clinical speech–based markers for mood disorders will help to ensure the ongoing development of this field and mitigate some of the risks and challenges highlighted from this review.

### Implications for Practice

The findings in this review suggest there are key opportunities for smartphone-based voice monitoring systems in the assessment and management of mood disorders. By linking the data generated by these monitoring systems, we may be able to deliver interventions at the right time, when care is most useful and crucial for the individual. This would prove beneficial as face-to-face therapeutic interventions are primarily based on retrospective and subjective information, and evidence suggests that mental health disorders can become increasingly difficult to treat the longer it is left untreated. However, there is a need to consider the limitations of the current technology. A review by Dogan et al [28] stated that relying on mental health apps for disorder management and therapy would be placing false trust and confidence in a young technology, and that a broader empirical database is needed regarding effectiveness and potential adverse effects of continuous monitoring of physiological and behavioral data using smartphone devices.

Whilst smartphone-based voice data collection provides a level of objectivity in the detection and monitoring of mood disorders, these data cannot currently be used alone in clinical management—these technological tools should be considered as “add-ons” that support practitioners to detect early signs of relapse and remission.

Although there is still skepticism about the potential of smartphones to provide meaningful data to help detect and monitor mental illness, uncertainties are starting to reduce due to the success of modern machine learning methods [13]. Further research demonstrating whether this can be a robust, cost-effective, and acceptable approach is needed before a clear transition into clinical practice can be made.

Additionally, despite the high prevalence of depression, mental health service access remains suboptimal and there remain gaps between service capacity and the needs of the general population. This is likely to be exacerbated by the increasing psychological distress reported globally [61], which has posed considerable pressures on the health care system. New methods of diagnosing and monitoring mood disorders will not only ameliorate the considerable demand placed on mental health services but also potentially allow wider access to mental health interventions [62].

### Limitations

This review has 4 key limitations. First, this review did not focus on the ethical and acceptability aspects of smartphone-based monitoring due to the limited data available on these aspects. This is a key area that future research should focus on as it affects the feasibility, acceptability, adherence, and ultimately uptake of these technologies in practice, and thus are crucial barriers to the successful implementation of smartphone-based monitoring into routine practice. As more data on acceptability are reported, future reviews should focus on this to aid decision makers on the clinical translation of these advances.

Second, restrictions in the search methodology may have resulted in relevant articles being missed, for example, the exclusion of gray literature and broad search terms. This is a common limitation reported in scoping reviews, attributable to the balance between achieving both breadth and depth of analysis within a rapid timeframe [63]. This review was successfully able to map a broad cross-section of the literature and provide a useful synthesis for researchers, engineers, and clinicians to understand the potential and technical feasibility of smartphone use and machine learning within their respective fields. Although a more comprehensive systematic review would provide greater clarity on gaps in the literature (in terms of possibilities of this methodology to differentiate mood states and the accuracy/practicalities/feasibility to implement them in real-world clinical practice), such a review would be less feasible to complete and would quickly be out of date given the rapidly evolving nature of the field. Further to this point, the search string used to identify relevant articles was too broad, as most of the included studies were identified through reference lists and review searching. This could be attributed to the nonstandardized definition of the concept of speech patterns. For instance, while this review refers to “speech features” or “patterns,” the term varies across the literature, for example, “vocal cues” [23] “the acoustics of speech,” [48] and “voice features” [28]. As the field continues to develop, this concept will need to be homogenized in order to improve the quality of review findings.

Third, the review was limited only to one possible digital measure of mood disorders—voice data. There are other features such as heart rate variability and physical activity that can be used to detect mood changes which were not explored in this review. Speech characteristics is however one of the key symptoms of mood disorders. Yet, speech as a digital domain has received relatively less attention than others. This review synthesizes the current evidence to provide clinicians and researchers a summary of which speech features are measurable and the technical considerations in assessing these, which can be used to inform future software development for voice analysis. There remain information gaps and challenges to enable transition of this technology into clinical practice.

### Conclusions

The aim of this review was to synthesize the state of research on voice analysis from smartphones to diagnose and monitor mood disorders. Findings from this synthesis may have implications for the development of speech-based classification systems for smartphones which may allow early identification of behavioral markers of mental health disorders so that health care providers can react early to patients’ needs and deliver timely and personalized treatment. While several research groups have started developing smartphone-based tools for the diagnosis and monitoring of mood disorders and have produced promising tests of feasibility, this review highlights that only a small number of systems that are currently available or are in preparation have been subjected to empirical studies. Nonetheless, smartphone-based monitoring of objective data in mood disorders is a rapidly growing approach and a highly innovative research field. This is evident in a number of study protocols stating ambitions to expand and intensify research in the field [64,65]. Although promising, a much larger evidence base is required to fully realize the potential, as well as the risks, of these approaches.

## Appendix

Multimedia Appendix 1 Detailed search strategy applied to all Databases.

## Abbreviations

BD: bipolar disorder
EMA: ecological momentary assessment
GFCC: gammatone frequency cepstral coefficient
GMM: Gaussian mixture model
HNR: harmonic-to-noise ratio
IVR: interactive voice response
LPCC: linear prediction cepstral coefficient
MFCC: Mel-frequency cepstral coefficients
PRISMA: Preferred Reporting Items for Systematic Reviews and Meta-Analyses
PTSD: post-traumatic stress disorder
SC: spectral centroid
SD: secure digital
SF: spectral flux
SVM: support vector machine
TEO: Teager energy operation
