# Supplementary material for: Using Acoustic Speech Patterns From Smartphones to Investigate Mood Disorders: Scoping Review
Source: JMIR Mhealth Uhealth. 2021 Sep 17;9(9):e24352. doi: 10.2196/24352 (PMC8486998; doi:10.2196/24352)
Supplement: Multimedia Appendix 1 [file mhealth_v9i9e24352_app1.docx]

## Multimedia Appendix 1

## Detailed search strategy applied to all Databases

depress* OR mood disorder OR (mood AND disorder) OR mood disorder* OR affective disorder OR (affective AND disorder) OR affective disorder* OR bipolar disorder OR (bipolar AND disorder) OR bipolar disorder* OR major depressive disorder OR MDD OR major depression OR unipolar depression OR affective symptoms

AND

cell phone OR mobile phone OR smart phone OR smartphone OR smart-phone OR MHealth OR mhealth OR m-health OR M-health OR phone OR iPhone OR android OR mobile OR smartphone application* OR mobile health OR mobile sensing OR wearable OR biosensor OR biosensor* OR bio-sensor* OR biomedical sensor OR biomedical sensor* OR monitor OR sensing OR sensor OR early detection

AND

prosod* OR voice analysis OR voice analyses OR voice analys* OR speech analysis OR speech analyses OR speech analys* OR phonetic analysis OR phonetic analyses OR phonetic analys* OR voice sound characteristics OR speaking behavior OR vocal biomarker* OR speech OR speech rate OR phone calls OR voice OR vocal OR biofeedback OR voice data
